# Epidemic Cerebro-Spinal Meningitis

**Published:** 1905-04-15

**Authors:** 


### Epidemic Cerebro-Spinal Meningitis.

The reports describing the course of the outbreak of epidemic cerebro-spinal meningitis at present in progress in New York necessarily possess a considerable measure of medical interest, and in view of the social and commercial possibilities of the disease they are not without importance to the general community. When we learn that in one of the most important and highly organised cities of the world several hundreds of deaths have occurred in the course of a few weeks from an epidemic disease which in normal circumstances makes no contribution to the death-roll, we are obviously face to face with an event of great pathological and social importance. The interest is by no means lessened by the announcement that an outbreak of the same disease has occurred in Germany, and has obtained in one district a degree of prevalence sufficient to interfere with so imperious a claim as the manœuvres of an army corps. The scientific aspect of these events will doubtless be duly presented by the medical authorities more immediately concerned; and it is to be hoped that this more recent experience will add something to our knowledge of the disease in question, and more particularly will help to penetrate the mystery which shrouds the mode of its epidemic extension. The hope that this end will be attained rests very largely upon confidence in the improved bacteriological methods which have been developed since the date of former outbreaks. One of the peculiarities of epidemic cerebro-spinal meningitis has been the complete uncertainty of the mechanism of its spread. Direct transference from patient to patient seems to be decidedly uncommon, whilst it is fairly certain that neither food nor water acts as the carrying agent. Obviously the first step necessary to obtain control of the disease is to substitute knowledge for ignorance on this point, and doubtless resolute efforts will be made in this direction. It is announced from New York that a health census of the city is to be made, and it is to be hoped not only that the epidemic will be stayed, but also that the complete pathology of the disease will be discovered.

A question for the Government and people of Great Britain is whether or not an outbreak of the disease in this country is at all probable. There seems to be little doubt that, whilst propagation from person to person is exceptional, the virus may be carried by individuals from place to place. And in view of the abundant traffic between New York and British ports, there would appear to be ample justification for the fear that the disease may be expected on our shores. Happily, history, so far as it goes, may be quoted to disarm that fear. Epidemics of cerebro-spinal meningitis have numbered not a few in the hundred years or so which have elapsed since the disease was definitely identified. Yet from all of them Great Britain has almost wholly escaped. As on the present occasion, the United States and Germany have been the chief sufferers. On what our immunity depends it is difficult or impossible to say, and in view of this it would be clearly unsafe to conclude it will continue. Hence the health authorities of our ports will naturally keep a specially keen eye on arrivals from the infected countries. It is, however, certain that our escape from epidemic developments has not depended upon the successful exclusion of the virus. For whilst no considerable outbreak has occurred in Great Britain, occasional isolated sporadic cases have been reported, and in several instances series of several cases have been noted in the same locality or institution. Ireland has been less fortunate than other parts of the United Kingdom; but even there the disease has never attained the degree of epidemic prevalence which has marked its progress in Europe and America. The history of epidemic cerebro-spinal meningitis thus has for ourselves an element of reassurance, though it by no means affords a guarantee of safety. Sooner or later, doubtless, medical and sanitary science will succeed in penetrating the secret of the disease, and in preventing such fatal and alarming outbreaks as are at present forcing themselves on our notice.

